# Dietary inflammation: a potential driver of atopic dermatitis?–Evidence from KNHANES 2017–2023

**DOI:** 10.3389/fimmu.2025.1606145

**Published:** 2025-06-20

**Authors:** Kaiyue Tan, Nanren Sun, Dongyang Wang, Jiaojiao Chen, Jiaqi Long, Junbin Zhang

**Affiliations:** ^1^ The First Clinical College of Shandong University of Traditional Chinese Medicine, Jinan, Shandong, China; ^2^ Department of Dermatology, Affiliated Hospital of Shandong University of Traditional Chinese Medicine, Jinan, Shandong, China

**Keywords:** atopic dermatitis, dietary patterns, Dietary Inflammation Index, KNHANES, sex stratification, age stratification, restricted cubic spline models

## Abstract

**Background:**

The global incidence of atopic dermatitis (AD) has risen significantly in recent decades, with trends showing spatial and temporal coupling with dramatic changes in dietary habits during industrialisation. Although the Dietary Inflammatory Index (DII), a tool to quantify the inflammatory potential of diet, has made breakthroughs in the study of chronic inflammatory diseases, large-scale cross-sectional evidence for its association with AD is still lacking.

**Methods:**

Based on large-scale population-based cross-sectional data from Korean National Health and Nutrition Examination Survey(KNHANES)2017-2023, the association between DII quartiles and AD risk was analysed using weighted multivariate logistic regression, with adjusted odds ratios (aORs) and 95% CIs calculated, stratified by sex (male/female) and age (≤54 vs >54); interactions were assessed by the Wald test, and the association between dietary index and risk was assessed using the Restricted cubic spline(RCS) models (with nodes set at the 10th, 50th, and 90th percentiles of DII) were used to explore non-linear associations, with models adjusted for covariates such as sex, age, and education.

**Results:**

Higher DII scores showed a significant association with AD prevalence. Participants in the highest DII quartile had a 73% higher risk than those in the lowest quartile (aOR = 1.73, 95% CI = 1.45-2.07).and the sex interaction was significant (interaction p<0.05), with stronger associations in the female group; RCS analyses showed a possible linear association between DII and AD risk (non-linear p>0.05).

**Conclusion:**

High dietary inflammatory index was significantly and positively associated with high prevalence of AD, especially in female and younger age groups; Notably, higher intake of dietary fiber and carotene is associated with a lower prevalence of AD.

## 1 Introduction

AD is a chronic, relapsing, inflammatory skin disease characterised by dry, intensely itchy, chronic eczema-like lesions (1), the prevalence of which has shown a significant global increase in recent decades. According to the World Health Organization (WHO), the prevalence of AD among children in developed countries has climbed from 2-5% in the 1960s to 15-30% today, and even reached more than 30% in some high-income countries (e.g., the United States, Australia) (2). This suggests that AD has become a global public health challenge.

It is noteworthy that the rising incidence of AD shows a significant spatial and temporal coupling with the dramatic changes in dietary habits during industrialisation. A cross-national study found that countries with westernised dietary patterns had annual increases in AD prevalence of up to 2.8-4.1%, significantly higher than in areas with >50% retention of traditional diets (0.7-1.2%) (3). Modern dietary patterns are dominated by highly processed foods, refined sugars and saturated fats, accompanied by a sharp decline in dietary fibre and plant polyphenol intake (4, 5). This dietary shift leads to a 2.3-3.3 unit elevation in DII compared to traditional patterns such as the Mediterranean diet (6–8), creating a pro-inflammatory metabolic microenvironment that increases the prevalence of inflammatory diseases such as AD.

In recent years, there have been breakthroughs in research on the association between diet and chronic inflammatory diseases, including the landmark proposal of the DII. Developed by Shivappa et al. in 2014, the DII assesses the inflammatory potential of diets based on the pro-inflammatory and anti-inflammatory properties of various dietary constituents, and has been widely used to explore the relationship between diet and inflammation-associated disease outcomes (9). A clinical intervention trial showed a 29.7% reduction in Scoring Atopic Dermatitis(SCORAD) scores after 8 weeks of a low DII diet (rich in omega-3 fatty acids, flavonoids) in patients with AD (10), however, no large-scale cross-sectional study of DII and AD has been conducted, and there is a need for such a study to better understand the relationship between DII and AD. The aim of this study was to investigate the relationship between DII and AD in Korean residents using data from the KNHANES 2017-2023.

## 2 Methods

### 2.1 Design and data collection

**Figure 1:**
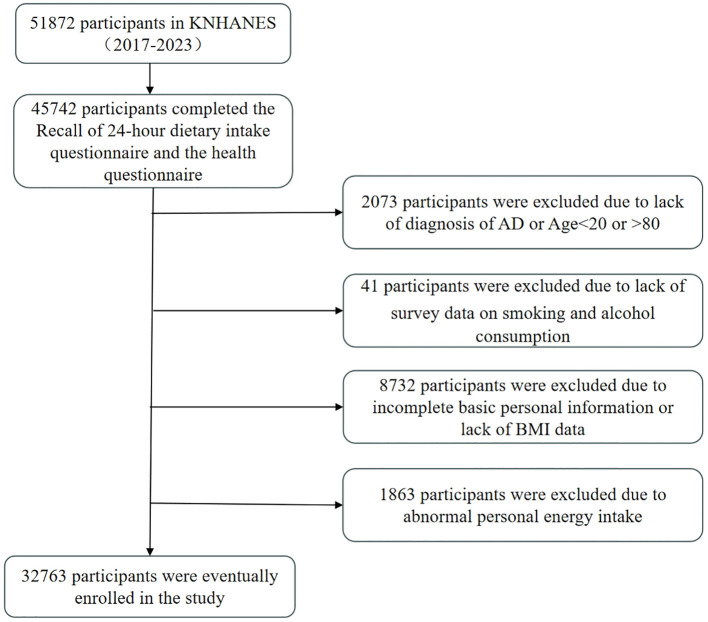
Study participant flowchart.

This study utilised data from the seven-year KNHANES from 2017 to 2023, which was conducted by the Korean Centers for Disease Control and Prevention. The objectives of the KNHANES include monitoring trends in health risk factors and their prevalence, and the survey consisted of health screenings, health interviews, and nutritional surveys conducted by trained medical staff and interviewers. The Institutional Review Board of the Korean Centres for Disease Control and Prevention approved the survey and all participants signed an informed consent form. This study was a secondary analysis of de-identified data from the KNHANES and therefore did not require additional ethical approval. Detailed information and description of the database can be found on the KNHANES website (https://knhanes.kdca.go.kr). For the 2017-2023 survey, a total of 51,872 individuals were sampled, of which 45,742 completed the 48-hour dietary recall (48RC) survey. Of the 45742 participants, we excluded the following participants: those who did not fully answer questions about AD and ages <20 and >80 years (n=2073); those who did not answer whether they had ever smoked or drank alcohol (n=41); those with incomplete personal information (did not fully answer questions about level of education, household income (quartiles), occupation, and exercise), and those who did not have a measured body mass index (BMI) (n=8732); in addition, we excluded participants with abnormal Energy intake (EK) (EK <500Kcal or EK >6000Kcal) (n=1863). Therefore, a total of 32,763 participants were eligible for our study (**Figure 1**).

### 2.2 Definition of covariates

We used the following variables as covariates: age (years), sex (male and female), area of residence (urban and rural), level of education (below university and university and above), household income (which will be categorised as low, medium, upper-middle, and upper based on quartiles), alcohol consumption (categorised as drinker and non-drinker based on current drinking or not), smoking (categorised as non-smoker, current smoker, and past smoker), exercise (categorised as no exercise, four and less, and five and more based on the number of number of days in a week that exercise (walking and strength) was performed was classified as no exercise, four or less, and five or more), and BMI calculated as weight in kilograms divided by the square of height in metres, based on the reference values for obesity in Asian populations (11), subjects were classified into three groups: underweight (BMI < 18.5), normal (18.5 ≤ BMI < 25), and obese (BMI ≥ 25).

### 2.3 Assessment of the Dietary Inflammation Index

In this study, the calculation of DII was based on an established standard model. The model first standardises the intake of the 45 dietary nutrients to the Z-score of the global average intake, then multiplies the standardised values with the corresponding inflammatory impact weights of each component, and finally adds all the weighted scores to give an individual’s total DII score. Higher DII scores indicate that the diet is pro-inflammatory, while lower scores reflect that the diet has anti-inflammatory potential (9).

**Figure 2:**
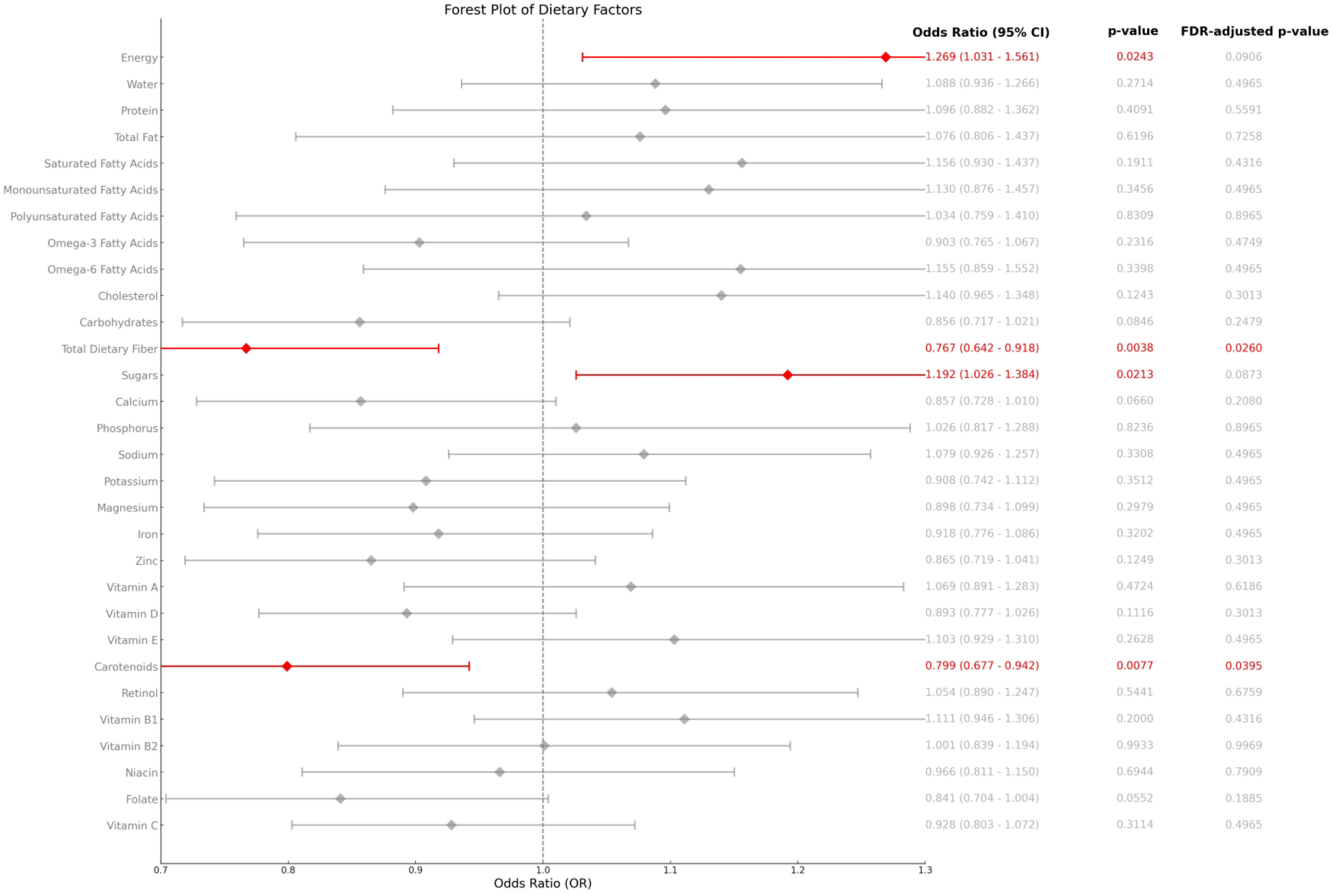
Relationship between components of the Dietary Inflammation Index score and AD.

Nutrient intake data in the study were obtained from KNHANES, where trained investigators obtained information on subjects’ food intake over the past 48 hours through a 1-hour dietary recall interview and calculated individual daily nutrient intakes by referring to the 10th edition of the Korean Food Composition Table by the Korea Rural Advancement Agency. Due to data availability constraints in KNHANES, we utilised a modified DII based on 25 nutrients (listed in **Figure 2**). Prior studies support the validity of this approach for population-level analyses (9).

### 2.4 Assessment of atopic dermatitis

The presence of AD was determined using a standardised approach validated for epidemiological studies. Participants were asked: “Have you ever been diagnosed with atopic dermatitis (eczema) by a physician?” Those responding “yes” were classified as AD cases. This approach of self-reported physician diagnosis is commonly used in large-scale population surveys and shares conceptual similarities with validated questionnaire-based assessments employed in major international studies such as the International Study of Asthma and Allergies in Childhood (ISAAC) (12, 13). Both methodologies have demonstrated reliability in population-based studies of allergic diseases (14).

### 2.5 Statistical analyses

We used the PROC SURVEY procedure in SAS software (version 9.4, SAS Institute Inc., Cary, NC, USA) and applied survey weights to account for the complex sampling design of KNHANES. The basic characteristics of the study population were described as weighted means (standard error, SE) for continuous variables and weighted percentages (weighted standard error, SE) for categorical variables. Significance tests for continuous variables were performed using analysis of variance (ANOVA), while categorical variables were assessed for differences between subgroups by the Rao-Scott chi-square test. Associations analysed by multinomial logistic regression models, two models were used for covariate control: (Model 1) a base-adjusted model (age, sex, education level, household income, occupation, place of residence); and (Model 2) a multivariate-adjusted model further controlling for the effects of lifestyle factors (alcohol consumption, smoking, exercise), BMI, and total energy intake; the present study analysed dietary inflammation by weighted multivariate logistic regression model to analyse the association between DII and disease risk, and stratified by sex (male/female) and age (≤54 vs >54 years), the aOR of DII quartiles for each subgroup was calculated, along with their 95% confidence intervals; the Wald test was used to assess the interaction of sex and age, and the interaction between the same DII level and the same DII level among subgroups was calculated by calculating the The Wald test was used to assess the interaction between sex and age, and the significance of the interaction was determined by calculating the log(OR) difference at the same DII level and its standard error among different subgroups; the nonlinear association between DII and disease risk was also explored using RCS with the nodes at the 10th, 50th, and 90th percentiles of the DII distribution, and all the models adjusted for socio-demographic covariates such as sex, age, and education. Results are presented as OR and 95% CI using two-sided tests with a significance threshold of p<0.05.

## 3 Results

### 3.1 Association analysis of DII with lifestyle and socio-economic status

**Table 1** presents the characteristics of the study participants according to DII quartiles. The data showed that higher DII scores were significantly correlated with increasing age, being male, having less than a college education, a non-smoking, non-alcoholic lifestyle, lower household income, more frequent strength training, less walking training, and lower BMI.

**Table 1:** Demographic and lifestyle characteristics of study participants in four groups of DII scores, KNHANES 2017-2023 (n = 32763).

DII group Covariate	Q1 (Lowest)	Q2	Q3	Q4 (Highest)	P-value
Age (years)	50.27 ± 0.19	49.09 ± 0.20	47.78 ± 0.21	45.93 ± 0.22	**<0.0001**
Sex					**<0.0001**
Male	62.97 (0.60)	54.04 (0.64)	46.57 (0.65)	36.11 (0.64)	
Female	37.03 (0.60)	45.96 (0.64)	53.43 (0.65)	63.89 (0.64)	
Residential area					0.6419
Urban	85.47 (0.42)	85.33 (0.42)	85.96 (0.40)	85.88 (0.40)	
Rural	14.53 (0.42)	14.67 (0.42)	14.04 (0.40)	14.12 (0.40)	
Education					**<0.0001**
≤ High school	50.72 (0.64)	53.53 (0.65)	55.26 (0.65)	60.42 (0.64)	
≥ College	49.28 (0.64)	46.47 (0.65)	44.74 (0.65)	39.58 (0.64)	
Occupation					**<0.0001**
Non-manual labour	75.47 (0.55)	76.41 (0.54)	79.62 (0.51)	81.13 (0.50)	
Manual labour	24.53 (0.55)	23.59 (0.54)	20.38 (0.51)	18.87 (0.50)	
Household income*					**<0.0001**
Q1 (Lowest)	20.09 (0.52)	22.55 (0.54)	23.93 (0.55)	28.13 (0.57)	
Q2 (Low)	23.30 (0.55)	24.93 (0.55)	25.67 (0.56)	25.84 (0.57)	
Q3 (Medium)	26.53 (0.57)	25.23 (0.56)	24.99 (0.56)	24.86 (0.56)	
Q4 (Highest)	30.08 (0.59)	27.29 (0.58)	25.41 (0.56)	21.17 (0.53)	
Smoking status					**<0.0001**
Non-smoker	49.69 (0.64)	56.36 (0.64)	58.91 (0.64)	63.95 (0.63)	
Current smoker	31.38 (0.60)	25.11 (0.56)	22.49 (0.55)	17.13 (0.49)	
Ex-Smoker	18.93 (0.53)	18.53 (0.52)	18.60 (0.53)	18.92 (0.53)	
Drinking status					**<0.0001**
Non-drinker	7.00 (0.29)	7.72 (0.31)	8.34 (0.32)	10.09 (0.35)	
Drinker	93.00 (0.29)	92.28 (0.31)	91.66 (0.32)	89.91 (0.35)	
Weight training					**<0.0001**
None	65.62 (0.62)	70.68 (0.60)	72.60 (0.59)	78.39 (0.55)	
≤4 days/week	22.19 (0.55)	19.77 (0.53)	19.55 (0.53)	15.83 (0.49)	
≥5 days/week	12.19 (0.42)	9.55 (0.38)	7.85 (0.36)	5.78 (0.30)	
Walking training					**<0.0001**
None	13.27 (0.43)	15.38 (0.46)	15.35 (0.45)	18.72 (0.49)	
≤4 days/week	34.83 (0.61)	36.48 (0.62)	37.02 (0.62)	36.57 (0.62)	
≥5 days/week	51.90 (0.64)	48.14 (0.64)	47.63 (0.64)	44.71 (0.64)	
BMI*					**<0.0001**
Underweight	3.14 (0.23)	3.65 (0.25)	4.50 (0.27)	5.81 (0.30)	
Normal	58.13 (0.64)	59.97 (0.64)	60.86 (0.63)	60.24 (0.63)	
Obese	38.73 (0.63)	36.38 (0.62)	34.64 (0.62)	33.95 (0.61)	

*Household income quartiles: Q1 (lowest), Q2 (low), Q3 (medium), Q4 (highest),Currency used to quantify income levels is the Korean won.

*BMI categories: Underweight (<18.5 kg/m²), Normal (18.5–24.9 kg/m²), Obese (≥25 kg/m^2^).

All results are weighted and presented as means ± SE (continuous) or % (SE) (categorical). Bold p-values indicate significance (p < 0.05).

### 3.2 Relationship between DII and AD

**Table 2** demonstrates the cross-sectional association between DII score and AD. Without adjusting for covariates, higher DII scores were significantly associated to AD status, participants in the highest DII quartile had 73% greater odds of having AD versus the lowest quartile (aOR = 1.73, 95% CI = 1.45-2.07). This positive association remained significant after adjusting for the basic life variable model and the multivariate model (OR 1.23, 95% CI 1.02-1.49, p trend = 0.03041, OR 1.25, 95% CI 1.03-1.51, p trend = 0.0213), with the risk of AD prevalence elevated by approximately 15% for each unit of elevated DII.

**Table 2:** Ratios and 95% confidence intervals for multivariate logistic regression of the association between DII and AD.

DII group Model	Q1	Q2			Q3			Q4		
	ORs	ORs (95%CIs)	p-value	DR	ORs (95%CIs)	p-value	DR	ORs (95%CIs)	p-value	DR
Model1	1.0 (ref)	**1.41(1.17-1.70)**	**0.0003**	1.105	**1.38(1.15-1.67)**	**0.0006**	1.065	**1.73(1.45-2.07)**	**<0.0001**	1.147
Model2	1.0 (ref)	**1.29(1.07-1.56)**	**0.0090**	1.114	1.13(0.94-1.37)	0.2023	1.072	**1.23(1.02-1.49)**	**0.0304**	1.158
Model3	1.0 (ref)	**1.30(1.07-1.57)**	**0.0071**	1.115	1.14(0.94-1.38)	0.1770	1.072	**1.25(1.03-1.51)**	**0.0213**	1.159

DR: Change in risk of disease per additional unit of DII.

Model 1 is unadjusted. Model 2 is sex, age, Residential area, Edu, Occupation and Household income adjusted. Model 3 was multivariate adjusted. Bold values are defined as significant at p values < 0.05.

### 3.3 Relationship between the components of DII and other nutrients included in the data in the survey and AD

**Figure 2** shows the associations between the components of the DII and other nutrients included in the survey data and AD. After adjusting for covariates included based on model3, analyses of the components of the DII and other nutrients included in the survey data showed that carotenoid and dietary fibre intake were negatively associated with DII scores, whereas total energy and sugar intake were positively associated with the prevalence of AD, and these results suggest that specific dietary components may play a unique role in the mechanisms of AD.

### 3.4 Sex and Age differences in DII effects on AD

**Table 3** demonstrates the association between DII scores and covariates and AD through stratified analyses by sex and age. The analyses showed that among males, higher DII scores, living in the countryside, and current smoking were risk factors and higher household income was a protective factor, whereas among females, living in the countryside and engaging in mental labour significantly reduced the risk of AD, and alcohol consumption, higher levels of education, and performing weight and walking training also significantly increased the risk of AD. In the lower age group, living in the countryside, higher level of education, and engaging in mental labour significantly reduced the risk of AD, whereas higher DII scores, living in the countryside, drinking alcohol, higher level of education, and doing weight and walking training significantly increased the risk of AD, whereas no significant results were found in the higher age group. The pro-inflammatory effects of DII and smoking predominated in men, whereas in women socio-behavioural factors (level of education, type of occupation) may influence AD indirectly through stress or lifestyle. The lack of significant associations in the higher age groups may be related to the role of immune senescence in weakening dietary inflammation (15).

**Table 3:** Ratios and 95% confidence intervals of multivariate logistic regressions of sex and age differences in the effect of DII on AD.

Variable (Reference)	Category vs Reference	Male aOR (95% CI)	Female aOR (95% CI)	≤54 years aOR (95% CI)	>54 years aOR (95% CI)
**DII Quartile (Q1)**	Q2 vs Q1	1.01 (0.84-1.21)	**1.51 (1.09-2.10)**	**1.46 (1.15-1.86)**	0.95 (0.54-1.65)
	Q3 vs Q1	1.08 (0.91-1.29)	**1.75 (1.27-2.39)**	**1.36 (1.07-1.73)**	0.84 (0.47-1.51)
	Q4 vs Q1	**1.27 (1.07-1.50)**	**2.02 (1.49-2.74)**	**1.67 (1.32-2.10)**	1.04 (0.59-1.84)
**Residential area (Rural)**	Urban vs Rural	**1.23 (1.06-1.42)**	**0.59 (0.44-0.80)**	**0.56 (0.42-0.74)**	1.15 (0.71-1.84)
**Household income** **(per quartile)**	Per quartile increase	**0.93 (0.88-0.98)**	0.98 (0.90-1.07)	0.99 (0.92-1.06)	0.91 (0.75-1.11)
**Education (≤High school)**	≥College vs ≤High school	0.95 (0.83-1.09)	**1.57 (1.29-1.92)**	**0.80 (0.68-0.94)**	0.95 (0.53-1.68)
**Occupation (Manual labour)**	Non-manual vs Manual	0.94 (0.81-1.10)	**0.50 (0.33-0.76)**	**0.56 (0.43-0.73)**	0.84 (0.52-1.36)
**BMI (Normal weight)**	Underweight vs Normal	0.91 (0.66-1.25)	1.18 (0.83-1.66)	1.06 (0.77-1.45)	0.52 (0.12-2.20)
	Obese vs. Normal	0.90 (0.79-1.02)	0.82 (0.65-1.03)	1.04 (0.88-1.22)	0.75 (0.50-1.11)
**Drinking status (Non-drinker)**	Drinker vs Non-drinker	0.89 (0.77-1.03)	**2.20 (1.53-3.16)**	**1.77 (1.11-2.82)**	1.07 (0.65-1.75)
**Walking training (None)**	≤4 days/week vs None	0.96 (0.80-1.15)	**1.38 (1.02-1.88)**	**1.73 (1.30-2.30)**	1.11 (0.67-1.84)
	≥5 days/week vs None	0.99 (0.83-1.18)	**1.57 (1.17-2.12)**	**2.09 (1.59-2.76)**	1.08 (0.65-1.81)
**Strength training (None)**	≤4 days/week vs None	0.93 (0.76-1.12)	**1.40 (1.11-1.77)**	**1.22 (1.02-1.45)**	0.81 (0.42-1.54)
	≥5 days/week vs None	0.99 (0.77-1.27)	0.81 (0.50-1.33)	1.16 (0.86-1.56)	0.75 (0.37-1.55)
**Smoking status (Non-smoker)**	Current smoker vs Non-smoker	**1.41 (1.04-1.91)**	**1.79 (1.26-2.54)**	0.92 (0.75-1.12)	1.71 (0.88-3.31)
	Ex-smoker vs Non-smoker	1.22 (0.95-1.56)	**2.08 (1.56-2.77)**	0.82 (0.67-1.02)	1.07 (0.67-1.70)

Interaction Test:

1.Sex stratification: Z = 2.60, p = 0.009.

2.Age stratification: Z = 1.74, p = 0.08.

Bold values are defined as significant at p values < 0.05.

### 3.5 Restricted cubic spline analysis

**Figure 3:**
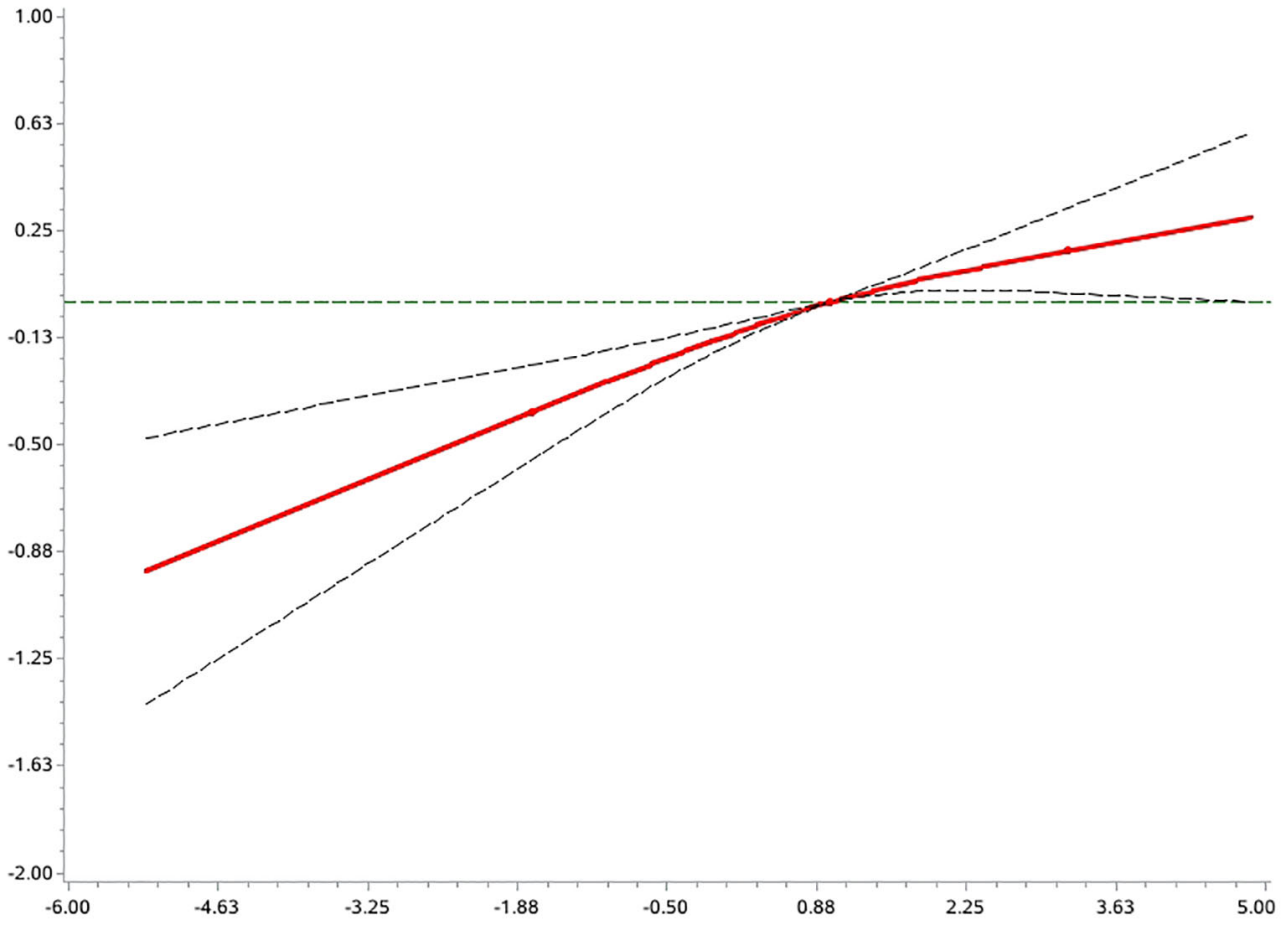
Non-linear analysis of DII and risk of AD prevalence.

An exploratory analysis of the potential non-linear association between the DII and the risk of AD prevalence was carried out using the RCS model (**Figure 3**). The results showed that the overall association between this variable and the outcome variable was statistically significant (p < 0.0001 for the overall test), and the non-linear component did not reach the level of significance (p = 0.18 for the non-linear test). This suggests a possible linear trend in the association between DII and risk of disease.

## 4 Discussion

In this cross-sectional study, elevated DII was associated with higher AD prevalence.

Further deconstruction of DII components showed that higher intake of dietary fiber and carotene is associated with a lower prevalence of AD, whereas total energy and sugar intake were associated with increased disease risk. Women in the highest DII quartile (Q4) showed twice the odds of AD presence compared to Q1 (OR = 2.02, 95% CI: 1.49-2.74),Men showed a more modest 27% increase (OR = 1.27, 95% CI: 1.07-1.50), An interaction test showed a significant interaction between DII and sex (Z = 2.60, p = 0.009), suggesting that women are more sensitive to the risk of a highly inflammatory diet. Participants ≤54 years exhibited significantly elevated risk with higher DII (Q4 vs Q1: OR = 1.67, 95% CI: 1.32-2.10);No significant association was observed in those >54 years (OR = 1.04, 95% CI: 0.59-1.84).The interaction approached statistical significance (Z = 1.74, p = 0.08), suggesting that there may be differences in response to inflammatory diets across age groups. Furthermore, analyses based on the RCS model showed that there was no significant non-linear relationship between DII and AD risk, which tended to be more linear. Therefore, the goodness of fit using a traditional multivariate logistic regression model also supports a robust effect of DII on AD risk. To the best of our knowledge, this study is the first to systematically assess the association between DII and AD risk in an Asian population, providing new epidemiological evidence in this area.

Previous studies have shown that higher DII scores are associated with increased levels of systemic inflammatory biomarkers such as interleukin (IL)-1, IL-4, IL-6, IL-10, C-reactive protein (CRP), and tumour necrosis factor-alpha (TNF-a) (16, 17), and that higher DII is associated with an increased risk of neoplasms, cardiovascular disease, metabolic disease, and others, which reveal an important role for anti-inflammatory diets in the treatment and prevention of disease (18–20). These findings reveal the important role of anti-inflammatory diets in disease treatment and prevention.

Studies have shown that anti-inflammatory dietary patterns (e.g., low DII) are negatively associated with AD risk (18–20), and several epidemiological studies have revealed significant associations between specific anti-inflammatory dietary patterns and AD risk: the Mediterranean diet has been shown to be protective due to its anti-inflammatory properties (e.g., rich in olive oil, fish, and fruits and vegetables), and studies have shown that its high adherence is associated with a reduced risk of AD (21); plant-based diets modulate gut flora through dietary fibre, and significantly reduce clinical symptoms and prevalence of AD (22, 23). A plant-based diet significantly reduces the clinical symptoms and prevalence of AD by regulating intestinal flora through dietary fibre metabolism. Modern diets, on the other hand, tend to be dominated by highly processed and modified foods, with a reduced intake of fruits and vegetables, which leads to an excessive intake of sugar and saturated fats (24). This dietary pattern is strongly associated with increased AD, as excess sugar and saturated fat may lead to elevated levels of inflammation in the body, which may trigger or exacerbate AD (25), these findings support the idea that dietary patterns influence AD pathology through modulation of inflammation and the microbiome, and provide a basis for the development of targeted dietary intervention strategies.

The core strength of this study lies in the systematic analysis of the association between DII and AD in the Korean adult population based on a large sample of data from a nationwide cohort and relying on standardised measurement methods for professionals. However, the study still suffers from the following limitations: DII calculations rely on a single 48-hour dietary recall, which carries the risk of daily fluctuations, recall bias, and social desirability bias, and may lead to misclassification of DII exposure (tending to weaken effect estimates). Inclusion of only 25 dietary components (out of the original 45) due to data limitations, omission of key anti-inflammatory components (e.g., flavonoids, allicin), and possible underestimation of true dietary inflammatory potential. Environmental exposures (PM2.5, allergens) (26) and atopic co-morbidities (asthma, rhinitis) (27) were not corrected for, which may interfere with DII-AD associations through socioeconomic factors or inflammatory pathways. Severity of AD (SCORAD) and age of onset data were lacking to distinguish the effect of diet on the onset, persistence, or worsening of AD. Cross-sectional studies could not determine the temporal relationship between DII and AD, and patients may change their diets in response to symptoms.

Future studies can reduce these problems through the following strategies: improving dietary assessment (using multiple 24-hour recalls or food frequency questionnaires (FFQs), validating DII scores in conjunction with inflammatory biomarkers (e.g., hs-CRP)), optimising DII modelling (incorporating missed anti-inflammatory components or developing Asian population-specific DIIs; quantifying the inflammatory potential of diets using metabolomics techniques), controlling for confounders (integrating environmental exposure databases (e.g., satellite remote sensing of PM2.5 data), detailed co-morbidity records, and medication use histories for mediator/effect modification analyses), deepening AD phenotypic analyses (collecting age of onset, severity indices, and recurrence frequency to distinguish differences in dietary effects of childhood/adult AD), and reinforcing causal inference (conducting prospective cohort (e.g., birth cohort tracking) or dietary intervention trials to clarify the temporal relationship between DII and AD).

In summary, this study demonstrated for the first time in an Asian population that high DII is positively associated with AD risk, and the effect was more pronounced in women and those ≤54 years of age. Despite some limitations-such as reliance on single dietary recall, simplified DII components, and uncorrected environmental exposures-the study’s nationally representative sample, standardised DII assessment, and stratified analyses provide a solid basis for conclusions. Future validation of associations through repeated dietary measurements, inclusion of omitted anti-inflammatory components, and prospective designs is warranted. Of note, the protective effects of dietary fiber and carotenoids consistently suggest that increasing whole grain/dark fruit and vegetable intake and decreasing refined foods could be an AD prevention strategy, especially for high-risk populations.

## Data Availability

The datasets presented in this study can be found in online repositories. The names of the repository/repositories and accession number(s) can be found below: https://knhanes.kdca.go.kr.
